# *ZcKr-h1* Knockdown Impairs Female Reproduction and Lifespan in *Zeugodacus cucurbitae* Under Normal and Short-Term High Temperature

**DOI:** 10.3390/insects17090920

**Published:** 2026-09-02

**Authors:** Zhenyao Qin, Shuyan Yang, Wenhong Xie, Ji Zhang, Yidan Lu, Longwei Wang, Wenjing Zhu, Shihao Zhou

**Affiliations:** 1 School of Tropical Agriculture and Forestry (School of Agricultural and Rural Affairs, School of Rural Revitalization), Hainan University, Danzhou 571737, China; qzy1878@126.com (Z.Q.); ysy000567@foxmail.com (S.Y.); xwhday100921@163.com (W.X.); you05263@126.com (Y.L.); 19808949354@163.com (L.W.); 2 Sanya Nanfan Research Institute, Hainan University, Sanya 572022, China; zhangji353797@163.com; 3 Wenchang Environment Monitoring Station, Wenchang 571300, China

**Keywords:** short-term high temperatures, melon fruit fly, *ZcKr-h1*, reproduction, fertility

## Abstract

Short-term high temperature is a critical environmental factor affecting the population dynamics of invasive insect pests. *Krüppel-homolog 1* (*Kr-h1*) is a key transcription factor involved in insect reproduction. In this study, we investigated the role of *ZcKr-h1* in female reproduction and lifespan under normal and short-term high-temperature conditions in the melon fly, *Zeugodacus cucurbitae* (Coquillett). RNA interference (RNAi)-mediated knockdown of *ZcKr-h1* markedly reduced female fecundity, shortened the oviposition period, delayed ovarian development, and decreased adult lifespan. Moreover, *ZcVgR* (vitellogenin receptor) expression was consistently suppressed following *ZcKr-h1* knockdown at both 25 °C and 45 °C, while *ZcVg3* expression was upregulated at 25 °C but showed no significant change at 45 °C. These findings demonstrate that *ZcKr-h1* is essential for female reproduction and survival under both normal and short-term high-temperature conditions, and that *VgR* is the primary downstream effector mediating its reproductive functions. This study provides a potential molecular target for environmentally friendly pest management during hot seasons.

## Simple Summary

Short-term high temperature is an important environmental factor affecting the population dynamics of invasive insect pests. *Kr-h1* is a key gene involved in insect reproduction and development. Here, we investigated the role of *ZcKr-h1* in female melon flies under normal and short-term heat conditions. We found that *ZcKr-h1* plays an important role in female reproduction and survival under both conditions. When we silenced this gene, egg production dropped sharply, the egg-laying period was shortened, ovarian development was delayed, and adult lifespan was significantly reduced. These findings demonstrate that although *ZcKr-h1* plays a critical role in regulating female reproduction and survival, additional factors may mediate the effects of short-term heat stress on *Vg3* expression.

## 1. Introduction

Temperature is known to be the most important environmental factor that affects the development, survival, reproduction, distribution, abundance, and population dynamics of insects [1]. In recent years, with the increase in the intensity and frequency of summer heatwaves, the non-lethal effects of short-term extreme high temperatures on insects have received growing attention [2,3]. Unlike constant high temperature, short-term high temperature can have specific and profound effects on insect reproduction, lifespan and population fitness [3]. These effects include reduced oviposition, shortened lifespan and impaired egg maturation [4,5]. Moreover, such adverse effects can be transmitted across generations, influencing offspring development and fitness [6]. However, at present, the understanding of the molecular mechanism of reproductive decline induced by short-term high temperature, especially the role of key transcription factors in this process, remains very limited.

Krüppel-homolog 1 (*Kr-h1*) is a transcription factor containing C_2_H_2_ zinc-finger domains. It was originally identified as a core downstream component of the juvenile hormone (JH) signaling pathway and plays a critical role in insect metamorphosis [7,8,9]. Recent studies have revealed that *Kr-h1* functions beyond metamorphosis. It also regulates ovarian development and vitellogenesis in the adult stage [10,11,12]. In *Nilaparvata lugens*, *Locusta migratoria*, *Bactrocera dorsalis* and several beetle species, silencing *Kr-h1* leads to marked downregulation of vitellogenin (*Vg*) and its receptor (*VgR*), arrested oocyte maturation, and a sharp decrease in oviposition [13,14,15]. Similar reproductive defects have also been reported in *Chilo suppressalis*, *Sogatella furcifera*, *Liposcelsis entomophila*, and *Coccinella septempunctata* [16,17,18,19]. Additional evidence indicates that *Kr-h1* is not only an executor of the JH signaling pathway but can also respond to external environmental signals. Additionally, in *Tribolium castaneum*, nutritional signals can regulate the expression of *Kr-h1*, thereby influencing yolk production [20]. In *Coccinella septempunctata*, low temperature and short light exposure can induce a decrease in JH titer accompanied by a significant downregulation of *Kr-h1* [21]. In *Colaphellus bowringi*, the environmental photoperiodic signal also regulates the switching between reproduction and diapause through the JH-MEt-Kr-h1 axis [22]. Given that low-temperature stress has been shown to downregulate *Kr-h1* expression, it is worth investigating whether short-term high temperatures, another form of extreme temperature stress, also influence reproductive output via *Kr-h1*. However, the effects of short-term high temperatures on reproduction through *Kr-h1* have not been experimentally validated in tephritid fruit flies (Diptera: Tephritidae).

The melon fly, *Zeugodacus cucurbitae* (Coquillett), is a globally invasive pest with a wide host range and high fecundity. It severely threatens cucurbit vegetable production [23,24,25]. Its adult emergence and oviposition peaks coincide with the hot, rainy summer season. Field populations are thus frequently exposed to short-term high temperature stress. After short-term high temperature treatment, adult survival declines, female oviposition decreases, and lifespan shortens. These negative effects can be transmitted to the next generation, reducing egg hatchability and pupation rate [26,27]. These heat-induced reproductive defects are consistent with phenotypes observed after *Kr-h1* silencing in other insects, suggesting that *Kr-h1* may act as a downstream executor of environmentally sensitive hormonal pathways. This suggests that short-term high temperature may impair reproductive output of *Z. cucurbitae* by affecting the normal expression or function of *ZcKr-h1*. Currently, control of *Z. cucurbitae* still relies on chemical pesticides, leading to increasing resistance and environmental concerns [24,25]. There is an urgent need to develop novel, environmentally friendly control strategies based on molecular targets. RNA interference (RNAi) can specifically suppress target gene expression at the post-transcriptional level and has been widely applied in functional studies and pest control target screening in many insects [28,29,30]. RNAi targeting *Kr-h1* has already shown reproductive suppression potential in *B. dorsalis* and other insects [13,15,16,17,18,19,22].

Based on the above analysis, this study investigated the function of *ZcKr-h1* in female reproduction and lifespan under normal and short-term high-temperature conditions. To verify this hypothesis, this study silenced *ZcKr-h1* using RNAi under normal temperature (25 °C) and short-term high-temperature simulated field heat wave (45 °C, 1 h) conditions. The system detected parameters such as the egg-laying quantity, egg-laying days, ovarian development and lifespan of female insects. In *Z. cucurbitae*, multiple vitellogenin genes have been identified [31]. Among them, *ZcVg3* was selected for analysis based on our previous studies, in which interfering with *ZcVgR* significantly affected *ZcKr-h1* and *ZcVg3* expression, while no significant effects were observed for the other Vg genes [32,33]. By analyzing the expression changes in ZcVg3 and *ZcVgR*, it clarified the reproductive regulation pathway of *ZcKr-h1* and its response to high temperature at the molecular level. This study aims to clarify how short-term high temperatures regulate the reproduction and survival of female fruit flies through *ZcKr-h1*, providing new insights into the regulation of insect reproduction under temperature stress and offering a theoretical basis for the RNAi control of fruit flies implemented as an auxiliary measure during the high-temperature season.

## 2. Materials and Methods

### 2.1. Insect Rearing

A laboratory population of *Z. cucurbitae* was established from adults collected from a bitter gourd field in Danzhou, Hainan Province (109°29′ E, 19°30′ N) and maintained in the laboratory for multiple generations before experiments. Larvae were reared on an artificial diet containing pumpkin, corn flour and yeast powder [26]. Adults were fed a 1:1 mixture of sucrose and yeast powder. Rearing conditions were 25 ± 1 °C, 70 ± 5% relative humidity, and a 14:10 h light:dark photoperiod. Newly emerged female adults were used for experiments.

### 2.2. Short-Term High Temperature Treatment

Based on field high-temperature conditions [34], newly emerged adults were subjected to a 1-h heat treatment at 25 °C (control) or 45 °C in a climate chamber (Hangzhou Qianjiang Instrument Equipment Co., Ltd., Hangzhou, China). No food was provided during the 1-h exposure, but a water dish was included to maintain humidity. After treatment, adults were returned to standard rearing conditions for subsequent experiments [34].

### 2.3. Design and Preparation of dsRNA

Double-stranded RNA (dsRNA) targeting *ZcKr-h1* was synthesized based on the sequence deposited in GenBank under accession number XM_011195543.3. Based on this sequence, primers for dsRNA synthesis were designed. The sense strand primer was 5′-taatacgactcactatagggGCGTATATGGAGCTGCGTTA-3′. The antisense strand primer was 5′-taatacgactcactatagggCACACTTGTACGGTCGTTCT-3′. T7 promoter sequences were added to the 5′ ends. The primers were used to amplify a 498-bp target region. The PCR amplification product was verified by agarose gel electrophoresis (Figure S1). The purified PCR product was used as the template for in vitro transcription using a T7 RNA Transcription Kit Plus (SJ001V2, Bolang Bio, Shanghai, China). The transcription reaction was carried out at 37 °C, followed by annealing at 72 °C for 10 min and cooling at room temperature for 20 min to form dsRNA. The dsRNA product was verified by 1% agarose gel electrophoresis and quantified by Nanodrop spectrophotometry (Thermo Fisher Scientific, Waltham, MA, USA; A260/A280 = 2.15).

### 2.4. Injection of dsRNA

Only female adults were injected. Newly emerged adults were placed in climate chambers and subjected to one of two temperature treatments: 25 °C (control) or 45 °C. Both treatments lasted 1 h. After treatment, adults were transferred to normal rearing conditions and provided with artificial diet until 6 days of age. Before injection, dsRNA was diluted with Tris-EDTA (TE) buffer (Phygene, Fuzhou Feijing Biotechnology Co., Ltd., Fuzhou, China) to 10 µg/µL. Using a stereomicroscope (Olympus SZX7, Tokyo, Japan), we identified the injection site as the inter-segmental membrane between the second and third abdominal tergites of the female. A microinjection needle loaded with the diluted dsRNA was mounted on an IM-11-2 manual microinjection pump (Narishige, Tokyo, Japan). A volume of 1.5 µL dsRNA solution was slowly injected into each female. Corresponding control groups were established: a blank control group (CK, without injection) and a negative control group (NC, injected with Tris-EDTA (TE) buffer). Each treatment had three biological replicates, with 10 females and 10 males per replicate.

### 2.5. qRT-PCR Analysis

The GenBank accession numbers for the target and reference genes are as follows: *ZcVg3*, XM_011186836.3; *ZcVgR*, XM_011179026.3; *ZcSDHA*, XM_011193311.3. Total RNA was extracted using an Animal Tissue/Cell Total RNA Extraction Kit (TSP413, Tsingke, China). For each replicate, two 6-day-old females were pooled for each replicate, at 24 h post-injection (i.e., 7 days post-eclosion). Three replicates were set. After concentration normalization, 12 µL (500 ng–2 µg) of total RNA was used for reverse transcription.

cDNA was synthesized using Goldenstar RT6 cDNA Synthesis Mix (TSK314S). First, 12 µL RNA was mixed with 4 µL 4× gDNA wiper Mix in a nuclease-free microcentrifuge tube. The mixture was incubated at 42 °C for 2 min to remove genomic DNA. Then, 16 µL of the DNA-removed reaction solution and 4 µL 5× qRT Super Mix II were added. Reverse transcription was performed at 50 °C for 15 min, followed by 85 °C for 2 min to terminate the reaction. The resulting cDNA was diluted with 80 µL ddH_2_O and mixed thoroughly.

The qPCR reaction mixture (10 µL) contained 5 µL 2× qPCR mix (SYBR Green I) (Tsingke Biotechnology Co., Ltd., Beijing, China; Cat. No. TSE202), 0.25 µL forward primer (10 pmol/µL), 0.25 µL reverse primer (10 pmol/µL), 2 µL cDNA template, and 2.5 µL ddH_2_O. qRT-PCR was performed on a QuantStudio™ 5 Real-Time PCR System (Applied Biosystems, Foster City, CA, USA). The thermal cycling program was 95 °C for 30 s; 40 cycles of 95 °C for 10 s and 60 °C for 60 s; followed by 95 °C for 15 s. A post-amplification melt curve analysis was performed, and a single peak was observed for each sample, confirming the specificity of amplification (Figures S2 and S3). The primers used are listed in Table 1. Three technical replicates were performed for each cDNA sample. *Succinate dehydrogenase flavoprotein* (*SDHA*) served as the reference gene [35]. Relative gene expression was calculated using the 2^−ΔΔCt^ method.

### 2.6. Effect of Silencing ZcKr-h1 on Reproduction and Lifespan of Z. cucurbitae (Coquillett)

After microinjection, adults from all groups were maintained under standard indoor conditions. Previous studies have shown that eggs laid by female *Z. cucurbitae* within the first 90 days of life account for more than 70% of their total lifetime egg production. Therefore, for each treatment group we recorded the number of eggs laid within the first 90 days, the number of egg-laying days during that period, ovarian diameter, and female lifespan. Female lifespan was defined as the number of days from eclosion to natural death and was recorded daily until all individuals had died. The 90-day period was used only as the egg-counting endpoint; survival was followed until death. For dsRNA-treated groups, which had significantly shorter lifespans (63–82 days), the 90 day count captures their entire lifetime fecundity. For control groups, the 90 day count represents >70% of expected lifetime output. Even under a conservative assumption that control groups could have laid 30% more eggs after day 90, the observed differences between treatments are of such magnitude that the incomplete observation window does not qualitatively affect the conclusions.

### 2.7. Statistics

Data were analyzed using Excel 2020 (Microsoft, Redmond, WA, USA) and SPSS 20.0 (IBM, Armonk, NY, USA). One-way ANOVA followed by Tukey’s HSD multiple comparison test and Student’s *t*-test were used for statistical analysis. Data are presented as mean ± standard error (SEM). Statistical significance thresholds are indicated in the corresponding figure legends.

## 3. Results

### 3.1. Effects of ZcKr-h1 Silencing on ZcVgR and ZcVg3

**Figure 1:**
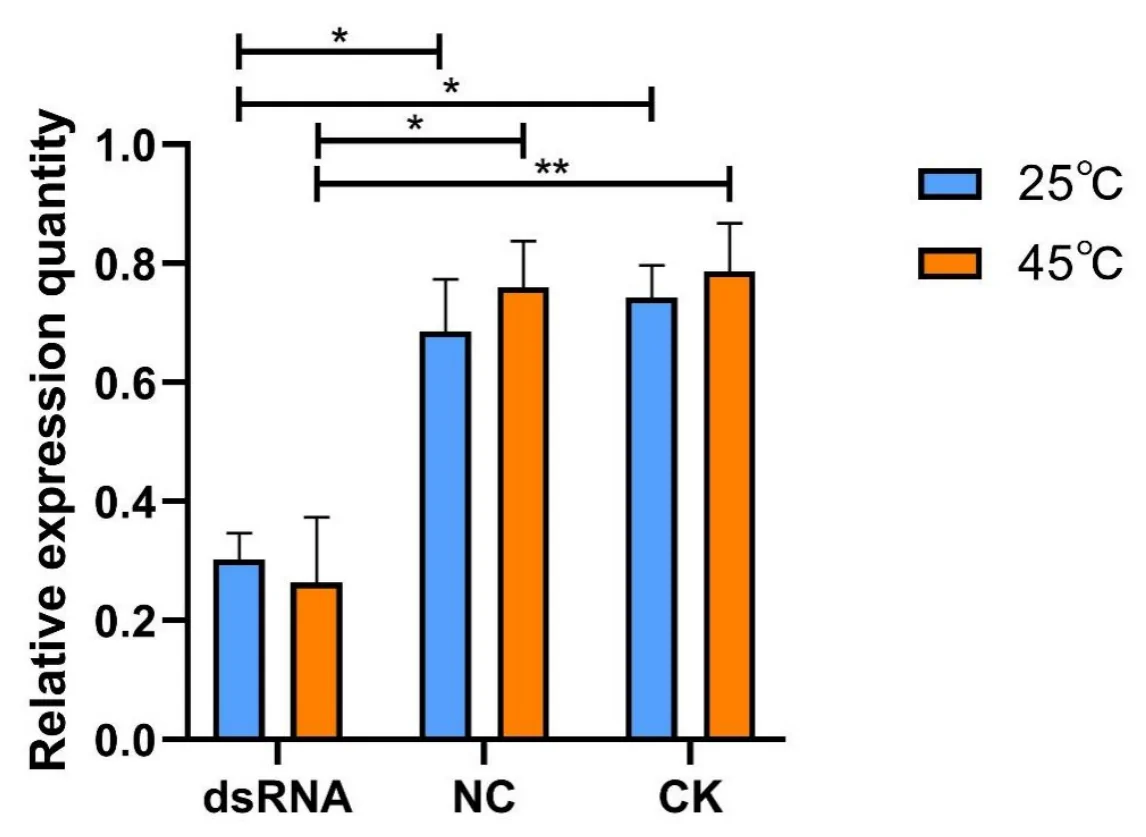
Knockdown efficiency of *ZcKr-h1*. Asterisks above bars indicate significant differences between groups (* *p* < 0.05, ** *p* < 0.01, Student’s *t*-test). Error bars represent SEM.

After the injection of ds *ZcKr-h1*, the expression level of the target gene decreased significantly (Figure 1). Under the condition of 25 °C, the expression level of the dsRNA group was 55.9% and 59.2% lower than that of NC and CK respectively. Under the condition of 45 °C, it decreased by 65.4% and 66.6% respectively, indicating that effective silencing was achieved at different temperatures.

**Figure 2:**
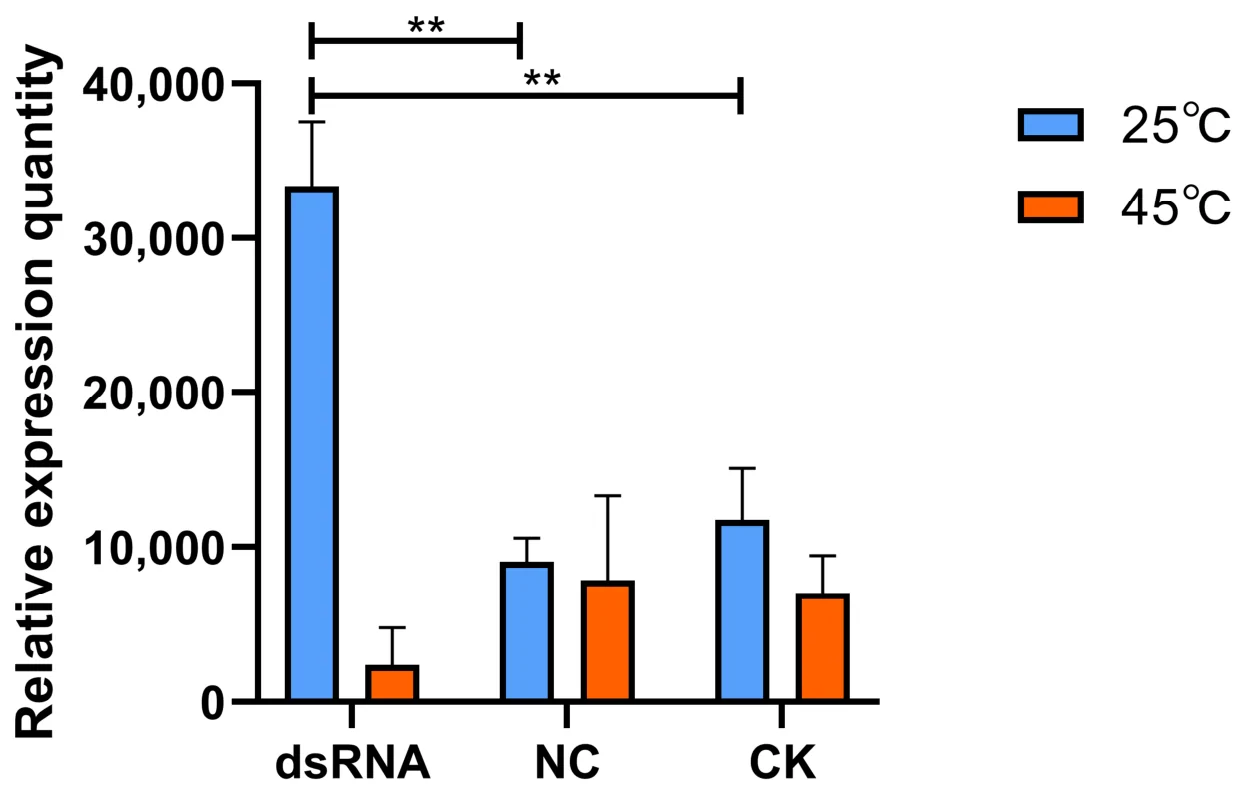
Effect of *ZcKr-h1* knockdown on *ZcVg3* expression. Asterisks above bars indicate significant differences between groups (** *p* < 0.01; Student’s *t*-test). Error bars represent SEM.

The effect of *ZcKr-h1* knockdown on *ZcVg3* expression was temperature-dependent (Figure 2). At 25 °C, *ZcVg3* transcript levels in the dsRNA group were approximately 269% and 184% higher than those in the NC and CK groups, respectively, suggesting that *ZcKr-h1* exerts a negative or indirect suppressive effect on *ZcVg3* under normal temperature. In contrast, after the 45 °C treatment, *ZcVg3* expression did not differ significantly among the three groups, indicating that heat stress overrides *ZcKr-h1*-mediated regulation and that *ZcVg3* expression may be governed by other pathways under thermal stress.

**Figure 3:**
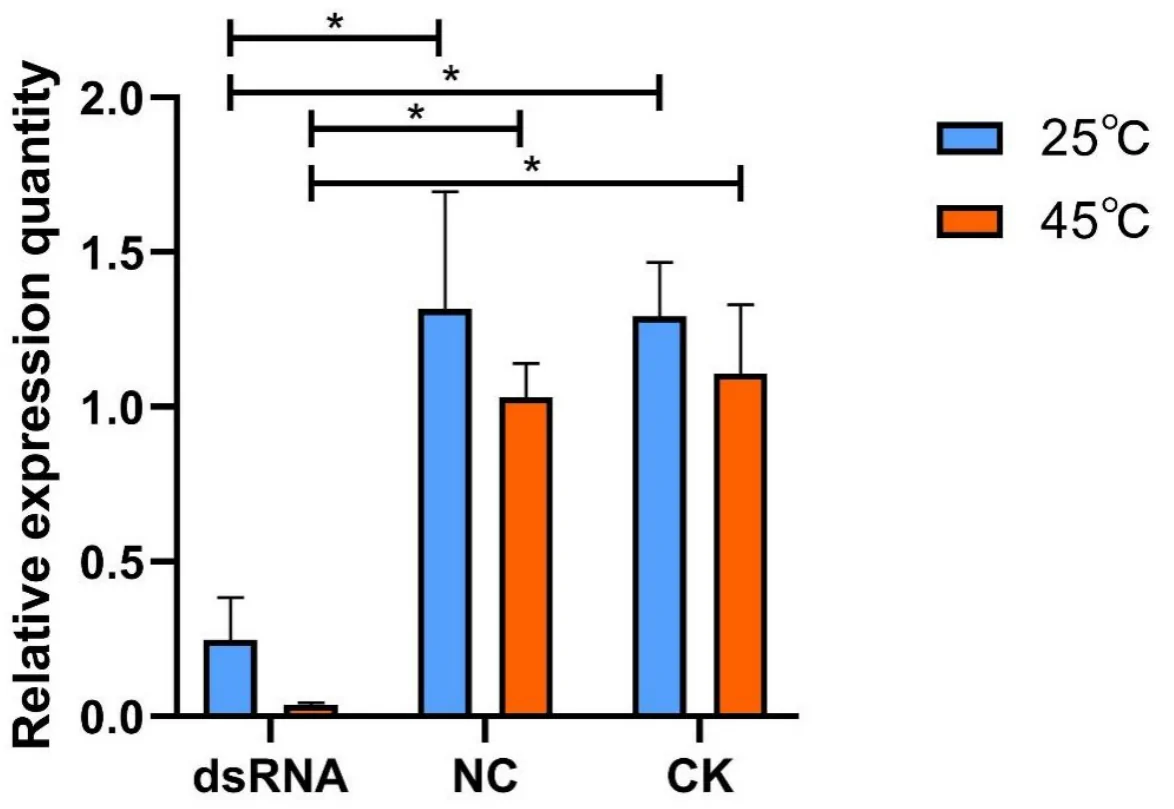
Effect of *ZcKr-h1* knockdown on *ZcVgR* expression. Asterisks above bars indicate significant differences between groups (* *p* < 0.05; Student’s *t*-test). Error bars represent SEM.

Unlike *ZcVg3*, *ZcVgR* expression was significantly suppressed by *ZcKr-h1* knockdown at both temperatures (Figure 3). At 25 °C, *ZcVgR* transcript levels in the dsRNA group were reduced by more than 80% relative to NC and CK; at 45 °C, the reduction exceeded 96%, demonstrating that *ZcKr-h1* functions as an essential positive regulator of *ZcVgR* and that this regulation is maintained under heat stress.

### 3.2. Effects of ZcKr-h1 Silencing on Reproduction and Lifespan

**Figure 4:**
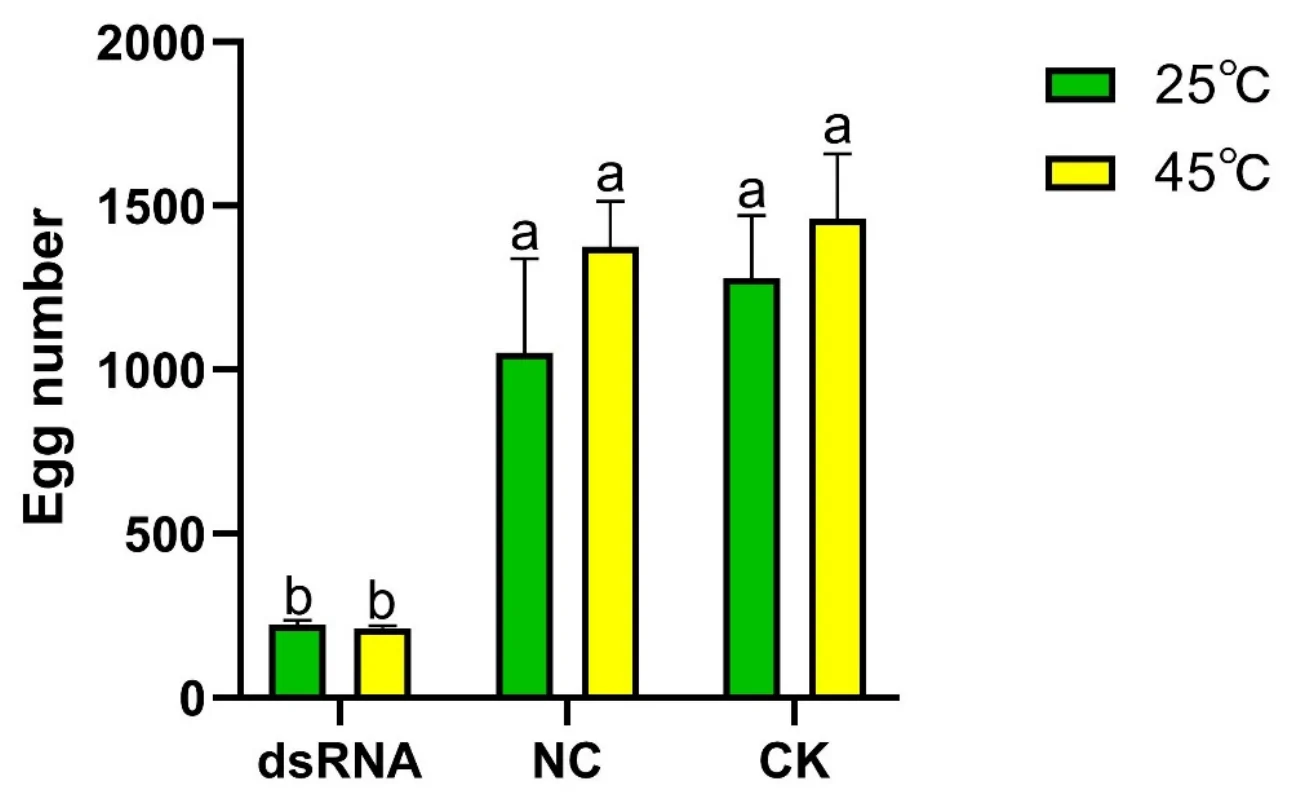
Effect of *ZcKr-h1* knockdown on fecundity. Different lowercase letters above bars indicate significant differences among treatment groups within each temperature (one-way ANOVA, Tukey’s HSD, *p* < 0.05). Error bars represent SEM.

*ZcKr-h1* knockdown substantially reduced the fecundity of *Z. cucurbitae* females (Figure 4). At 25 °C, dsRNA-treated females laid an average of 222.7 eggs, corresponding to 21.2% and 17.4% of the egg production recorded in the NC (1052.7) and CK (1281.0) groups. A similar pattern was observed at 45 °C, with dsRNA females laying only 212.7 eggs.

**Figure 5:**
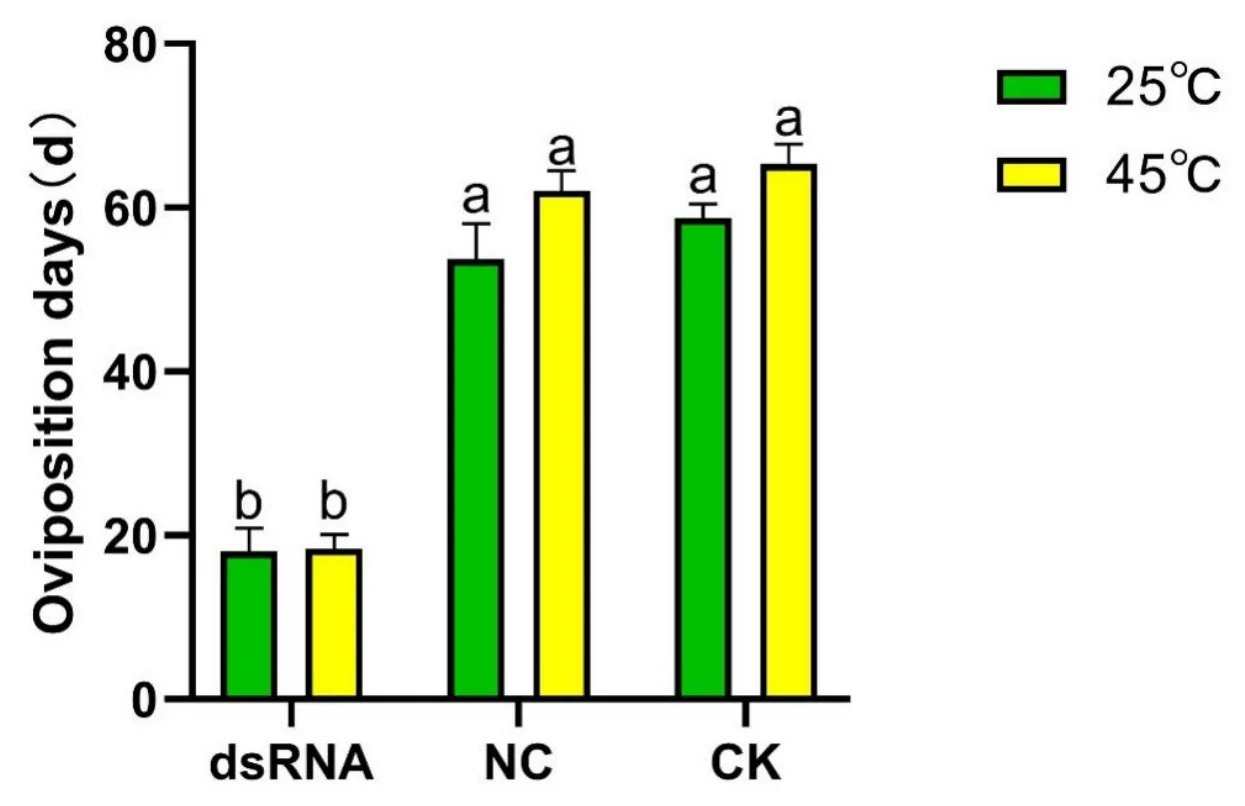
Effect of *ZcKr-h1* knockdown on oviposition days. Different lowercase letters above bars indicate significant differences among treatment groups within each temperature (one-way ANOVA, Tukey’s HSD, *p* < 0.05). Error bars represent SEM.

The oviposition period was also markedly shortened (Figure 5). At both 25 °C and 45 °C, dsRNA-treated *Z. cucurbitae* females laid eggs for only approximately 18 days, compared with 53–65 days in the control groups, indicating that loss of *ZcKr-h1* function severely truncates the reproductive window.

**Figure 6:**
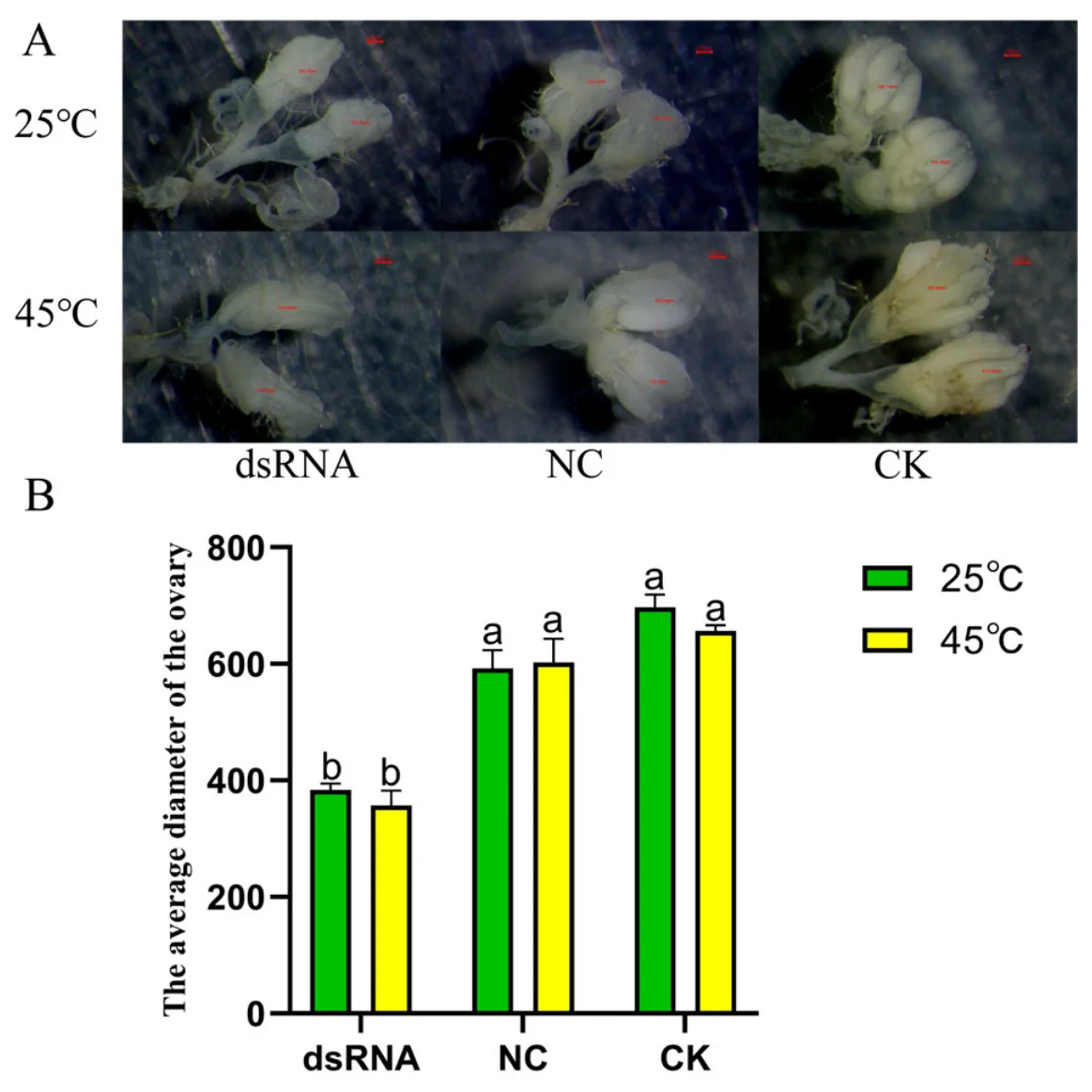
Effect of *ZcKr-h1* knockdown on ovary development. (**A**) Representative images of ovaries; red scale bar = 100 μm. (**B**) Average ovary diameter. Different lowercase letters above bars indicate significant differences among treatment groups within each temperature (one-way ANOVA, Tukey’s HSD, *p* < 0.05). Error bars represent SEM.

Ovarian development of *Z. cucurbitae* females was severely impaired by *ZcKr-h1* knockdown (Figure 6). The mean ovarian diameter of dsRNA-treated females (357–384 μm) was approximately half that of the controls (593–697 μm). The reduced ovary diameter indicates that *ZcKr-h1* silencing markedly slows ovarian development and further suppresses reproduction in female *Z. cucurbitae*.

**Figure 7:**
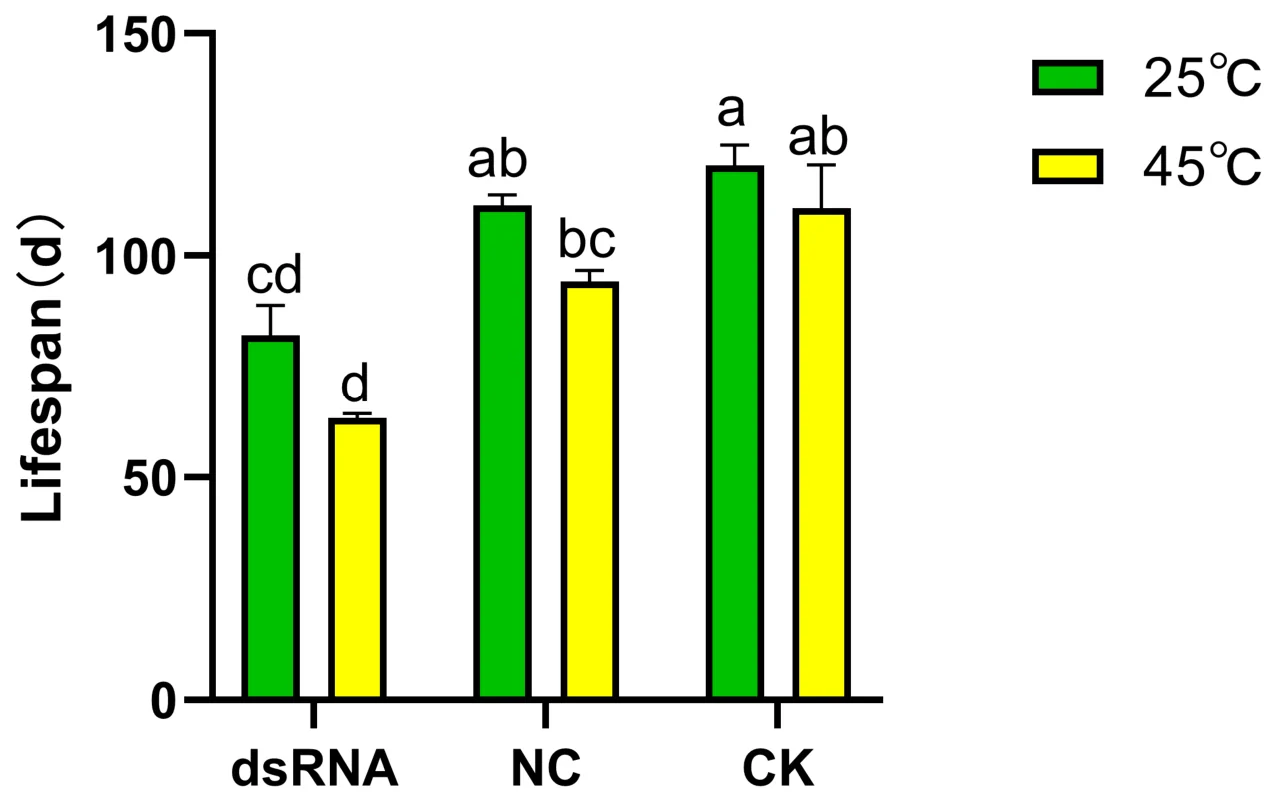
Effect of *ZcKr-h1* knockdown on lifespan. Different lowercase letters above bars indicate significant differences among treatment groups within each temperature (one-way ANOVA, Tukey’s HSD, *p* < 0.05). Error bars represent SEM.

*ZcKr-h1* knockdown also significantly reduced the lifespan of *Z. cucurbitae* females (Figure 7). At 25 °C, dsRNA-treated females lived an average of 82.0 days, compared with 63.3 days at 45 °C; both values were significantly lower than those of the respective controls. The additive effect of heat stress and *ZcKr-h1* knockdown on longevity suggests that *ZcKr-h1* contributes not only to reproductive regulation but also to adult somatic maintenance and survival.

## 4. Discussion

Insects in natural environments are more frequently exposed to short-term heat extremes than to sustained warming [3]. In this study, although the mean fecundity of control females exposed to 45 °C was numerically slightly higher than that of the 25 °C controls, this difference was not statistically significant (Figure 4). This numerical trend is in the same direction as some previous observations suggesting that short-term high temperature can stimulate oviposition in *Z. cucurbitae* [27,34]; however, under our experimental conditions, this effect did not reach statistical significance.

*Kr-h1* is a key transcription factor in the juvenile hormone signaling pathway and has a conserved role in insect growth, development, and reproduction [12]. In this study, we used RNA interference to silence *ZcKr-h1*, an approach previously applied in *Spodoptera exigua* [36], *Diabrotica virgifera* [29], *B. dorsalis* [30], and *Z. cucurbitae* [35]. Our results show that under the 45 °C short-term high-temperature condition, silencing *ZcKr-h1* was highly effective and led to a significant decrease in female oviposition and oviposition days. This directly confirms the critical role of *ZcKr-h1* in the reproductive regulation of *Z. cucurbitae* and is highly consistent with findings on *Kr-h1* in other insects. For example, silencing *BdKr-h1* in *B. dorsalis* obstructed ovarian development and drastically reduced reproductive capacity [13]; in *Liposcelis entomophila*, RNAi-mediated silencing of *Kr-h1* significantly prolonged the pre-oviposition period, severely impaired ovarian development, and reduced fecundity. These results therefore reinforce the functional conservation of *Kr-h1* in female insect reproduction.

A novel finding of this study is the differential regulation of two downstream targets by *ZcKr-h1*. Under normal temperature, *ZcKr-h1* knockdown unexpectedly upregulated *ZcVg3* while suppressing *ZcVgR*, suggesting that *VgR* is likely the primary downstream effector mediating the reproductive functions of *ZcKr-h1*, while *Vg3* may be regulated through additional compensatory mechanisms. The loss of *ZcVg3* differential expression under heat stress suggests that the regulatory effect of *Kr-h1* on *Vg3* is temperature-dependent and may be overridden by heat-activated pathways. In particular, exogenous *JH* treatment in a *ZcKr-h1*-silenced background should be performed in future studies to directly test whether *JH* regulates *ZcVg3* through *Kr-h1* or through alternative pathways. Under normal temperature, *ZcKr-h1* knockdown upregulated *ZcVg3* by approximately 2.7-fold while suppressing *ZcVgR* by over 80%; under heat stress, the *ZcVg3* difference among groups disappeared, yet *ZcVgR* remained consistently and profoundly downregulated regardless of temperature. Such differential regulation is rarely reported in existing *Kr-h1* functional studies. Since *VgR* is the sole receptor mediating vitellogenin uptake from the hemolymph into developing oocytes, its downregulation directly blocks yolk accumulation, whereas the upregulation of *ZcVg3*—even if it increases *Vg* precursor synthesis—cannot compensate for the uptake defect. A similar phenotype of deficient yolk deposition in oocytes has been observed in two *Henosepilachna* species following *Kr-h1* RNAi [14]. These results suggest that the critical reproductive output of *Kr-h1* is likely *VgR* rather than *Vg*—an important finding of the present study.

In addition to reproductive impairment, *ZcKr-h1* knockdown significantly shortened female lifespan, with heat stress and gene silencing exerting additive effects. This indicates that *ZcKr-h1* also contributes to adult survival. As a core transcription factor in the JH pathway, *Kr-h1* not only regulates metamorphosis and reproduction but may also modulate metabolic rate, immune function and stress responses. The significant lifespan reduction after silencing *ZcKr-h1* suggests that this gene has broader biological functions beyond reproductive control. In female insects, many studies have demonstrated that *Kr-h1* is required for vitellogenin expression; silencing *Kr-h1* by RNAi markedly reduces *Vg* transcript levels [15]. Furthermore, *Kr-h1* triggers polyploidization of fat body cells to meet the high protein synthesis demands during reproduction [10,13]. When *ZcKr-h1* expression is suppressed, yolk protein synthesis is blocked and fat body polyploidization is likely disrupted, compromising the overall physiological state and energy reserves of the adult and ultimately shortening lifespan. Therefore, targeting *ZcKr-h1* via RNAi offers a novel strategy for *Z. cucurbitae* management by simultaneously impairing reproductive capacity and adult survival. This finding provides a new molecular target and theoretical basis for developing environmentally friendly RNAi-based control methods for *Z. cucurbitae*.

A limitation of this study is the absence of a non-targeting dsRNA control such as dsGFP, which is generally recommended to exclude sequence-independent effects [37]. Although the blank control (CK) and Tris-EDTA (TE) buffer-injected negative control (NC) are still widely used as basic injection controls in insect RNAi studies [38], they cannot completely rule out sequence-independent off-target effects. Nevertheless, dsGFP itself may induce non-specific transcriptional responses in some insects, and its suitability can vary among species [39,40]. Therefore, future studies should include a species-appropriate non-targeting dsRNA control to further validate the specificity of the RNAi phenotype.

## 5. Conclusions

This study reveals that RNAi-mediated knockdown of *ZcKr-h1* markedly reduces fecundity, shortens the oviposition period, delays ovarian development, and decreases adult lifespan in *Zeugodacus cucurbitae* females. These phenotypic defects are accompanied by consistent suppression of *ZcVgR* transcription and, under normal temperature, upregulation of *ZcVg3*, indicating that *VgR* is the primary downstream effector mediating the reproductive functions of *ZcKr-h1*. Notably, heat stress and *ZcKr-h1* silencing exerted additive effects on lifespan reduction. Collectively, these findings establish *ZcKr-h1* as a key transcriptional regulator that couples thermal stress to reproductive output and somatic survival in a tephritid species. Targeting *ZcKr-h1* via RNAi therefore represents a promising strategy for the environmentally sustainable management of *Z. cucurbitae*, with potentially enhanced efficacy during high-temperature seasons.

## Figures and Tables

**Table 1:** Primer sequence information of target gene and reference gene.

Primer Name	Primer Sequences (5′ to 3′)	Use of Primers
*Succinate dehydrogenase flavoprotein*-F	TTGATTTCAAAATAGGCGCAGTG	References gene amplification in qPCR
*Succinate dehydrogenase flavoprotein*-R	CGATGGTACACGCATAAGGC	
*ZcVgR*-F	TGCTTTCCCGGTTATCGCTT	Target gene amplification in qPCR
*ZcVgR*-R	AACGTAATCGGTTGCTCCGT	
*ZcKr-h1*-F	GCCAAAGACTTACCAGACACT	
*ZcKr-h1*-R	TCGCCGCTTGAACTACTG	
*ZcVg3*-F	TTTGCTCCTCAGCACTCTCA	
*ZcVg3*-R	GCGCCATATTTGATCGGCA	

## Data Availability

The original contributions presented in the study are included in the article; further inquiries can be directed to the corresponding authors.

## Supplementary Materials

The following supporting information can be downloaded at: https://www.mdpi.com/article/10.3390/insects17090920/s1, Figure S1: Verification of the PCR amplification product used for dsRNA synthesis. Lane 1 DNA marker; Lane 2 PCR amplification product; Figure S2: qRT-PCR amplification curves. (a) *ZcKr-h1*, (b) *ZcVg3*, (c) *ZcVgR*, (d) *Succinate dehydrogenase flavoprotein* (*SDHA*). All samples showed successful amplification; Figure S3: qRT-PCR melt curve analysis. (a) *ZcKr-h1*, (b) *ZcVg3*, (c) *ZcVgR*, (d) *Succinate dehydrogenase flavoprotein* (SDHA). A single peak was observed for each primer pair, confirming primer specificity.
